# A systematic review and meta-analysis of risk factors for intensive care unit acquired weakness

**DOI:** 10.1097/MD.0000000000031405

**Published:** 2022-10-28

**Authors:** Zi Yang, Xiaohui Wang, Faying Wang, Zeyu Peng, Yuying Fan

**Affiliations:** a Clinical Nursing Teaching Department, The Second Affiliated Hospital of Harbin Medical University, Harbin, China; b School of Nursing, Harbin Medical University, Harbin, China; c Department of Critical Care Medicine, The Second Affiliated Hospital of Harbin Medical University, Harbin, China.

**Keywords:** evidence, incidence, intensive care unit acquired weakness, meta-analysis, risk factors, systematic review

## Abstract

The aim of this meta-analysis is to systematically evaluate and summarize the risk factors of intensive care unit acquired weakness (ICU-AW), to provide evidence-based evidence for the formulation of prevention strategies for ICU-AW.

**Methods:**

PubMed, EMBASE, Web of Science, CBM (China Biology Medicine, China), Chinese National Knowledge Infrastructure, Chinese WANFANG, and VIP will be searched to define relevant risk factors for ICU-AW. The databases search period is from January 1, 2005 to August 13, 2021. The Newcastle Ottawa Scale (NOS) is used to evaluate the quality of the included studies. RevMan 5.3 analysis software will be used for meta-analysis.

**Results:**

This systematic review and meta-analysis included a total of 12 cohort studies, including 9 international journals and 3 Chinese journals, with a total of 1950 patients, of which 856 had ICU-AW. The results showed that the significant risk factors for ICU-AW included female (odds ratio [OR] = 1.34, 95% confidence interval [CI]: 1.06–1.71; *P* = .02), mechanical ventilation days (OR = 3.04, 95% CI: 1.82–4.26; *P* < .00001), age (OR = 6.33, 95% CI: 5.05–7.61; *P* < .00001), length of intensive care unit (ICU) stay (OR = 3.78, 95% CI: 2.06–5.51; *P* < .0001), infectious disease (OR = 1.67, 95% CI: 1.20–2.33; *P* = .002), renal replacement therapy (OR = 1.59, 95% CI: 1.11–2.28; *P* = .01), use of aminoglucoside drugs (OR = 2.51, 95% CI: 1.54–4.08; *P* = .0002), sepsis related organ failure assessment (SOFA) score (OR = 1.07, 95% CI: 0.24–1.90; *P* = .01), hyperglycemia (OR = 2.95, 95% CI: 1.70-5.11; *P* = .0001).

**Conclusion:**

This meta-analysis provides comprehensive evidence-based on the assessment of the risk factors for ICU-AW, their multifactorial etiology was confirmed. This study indicated that female, mechanical ventilation days, age, length of ICU stay, infectious disease, renal replacement therapy, use of aminoglucoside drugs, SOFA score, and hyperglycemia are independent risk factors for ICU-AW. We have not found consistent evidence that corticosteroids, neuromuscular blockers, sepsis have any effect on ICU-AW risk.

## 1. Introduction

For the past few years, with the continuous development of critical care medicine and medical technology, the attention of medical staff has gradually shifted to the long-term impact of critically ill patients. In 2014, the American Thoracic Society drafted the intensive care unit acquired weakness (ICU-AW) guidelines,^[1]^ which were defined as: occurring during severe illness, not caused by severe disease, mainly manifested as clinical syndrome of new limb symmetry weakness. ICU-AW due to neuromuscular dysfunction is one of the serious complications in critically ill patients. In 1993, Ramsay^[2]^ proposed the concept of ICU-AW. Clinical manifestations include difficulty in weaning, paralysis or quadriplegia, decreased reflexes, and muscle atrophy. Some studies in the United States reported that the incidence of ICU-AW was 67%,^[3]^ and it was still as high as 36% after discharge.^[4]^ About the pathogenesis of ICU-AW is complicated and involves the functional and structural changes of muscles and nerves.^[5]^ ICU-AW can not only cause serious sequelae, including quadriplegia or paraplegia, but also cause permanent disability, which seriously affects the quality of life of patients after discharge from hospital.^[6]^ In view of the serious impact of ICU-AW on intensive care unit (ICU) patients, ICU-AW has become a hot research topic at home and abroad, attracting intensive attention from scholars at home and abroad. Relevant studies^[7,8]^ have shown that a more analytical classification is proposed to divide risk factors for ICU-AW into the several categories, namely general information and risk factors related to underlying diseases and risk factors related to treatment. But the current evidence is still inconclusive and there is no particularly effective treatment. Controlling risk factors may be the only preventive measure to reduce its incidence.^[9]^ Furthermore, the pathogenesis and mechanism of ICU-AW have not yet been fully clarified, and there is no effective treatment,^[10]^ Therefore, early identification of risk factors for ICU-AW is important. Early assessment of ICU-AW risk factors, and provide targeted interventions to achieve the optimal recovery goals of patients. Our objective is to provide an updated comprehensive systematic review of prospective studies on risk factors for ICU-AW, to identify the risk factors of ICU-AW, hoping to provide scientific reference for clinical development of ICU-AW prevention strategies.

## 2. Methods

### 2.1. Literature search and study selection

Systematic literature search and quantitative analysis were conducted according to the preferred reporting items in systematic review and Meta-analysis guidelines.^[11]^ This study did not require the approval of the ethics committee. Because all the data used for analyses were extracted from the published studies, the ethical approval and informed consent were not necessary.

### 2.2. Search strategy

As a basis for our analysis, PubMed, EMBASE, Web of Science, SinoMed, Chinese National Knowledge Infrastructure, Chinese WANFANG, VIP, and other databases will be searched systematically. The databases are searched from January 1, 2005 to August 13, 2021.

### 2.3. Literature inclusion and exclusion criteria

In this systematic review and meta-analysis, 2 researchers independently conducted a preliminary search according to the inclusion criteria and selected eligible studies. The inclusion are as follows: Literature type: domestic and foreign prospective cohort research literature published in Chinese and English, the topic is related the risk factors of ICU-AW; Research object: ICU adult patients (age ≥18 years old). The case group is ICU-AW patients, the rest are the control group, regardless of race, gender, or disease type; The diagnosis of ICU-AW: such as the Medical Research Council Scale or Nerve Conduction Studies (NCSs) and so on; Outcome indicators: the incidence of ICU-AW. The inclusion are as follows: Exclude studies that are inconsistent with the above conditions, repeated publications, incomplete information, gray literature dissertations, and data that cannot be extracted and used.

### 2.4. Data extraction

All studies were imported into EndNote X9. The 2 researchers read the title and abstract respectively according to the established inclusion and exclusion criteria for preliminary screening, further read the full text of the literature that meets the inclusion criteria for further screening to determine whether to include. Disagreements can be resolved by discussion or negotiation with third-party review (XBT). For each study, the 2 authors independently extracted data based on pre-designed table, including first author, publication year, country, sample size, incidence of ICU-AW, major risk factors, and diagnostic methods.

### 2.5. Assessment of study quality

The Newcastle-Ottawa Quality Scale (NOS)^[12]^ was used independently by 2 reviewers to evaluate the quality of the included literature. With a full score of 9 points, 0 to 4 is considered low-quality research and 5 to 9 is considered high-quality research. After the evaluation, the 2 researchers cross-check the results, discuss the inconsistencies or consult a third party to help judge.

### 2.6. Statistical analysis

Meta-analysis of the extracted data will be performed using Revman 5.3. For dichotomous variables, odds ratio (OR) and its 95% confidence interval (CI) were used as effect statistics, while for continuous variables, mean difference (MD) and its 95% CI were used as effect statistics. After data input, heterogeneity test is performed first. If *P* > .1 and *I*^2^ < 50%, the included data were considered to be homogeneous and the fixed-effects model was used for analysis. When *P* ≤ .1 and *I*^2^ ≥ 50%, sensitivity analysis was used to analyze the source of heterogeneity and calculate the comprehensive effect after excluding the studies that caused heterogeneity. If heterogeneity remains large, use a random-effects model or discard the combined results and use descriptive analysis. If *P* < .1, *I*^2^ ≥ 75%, and the heterogeneity is too large to determine the source of heterogeneity, the data will be described without merging. When the number of articles included in the analysis of a single risk factor was more than 10, the funnel plot was used to analyze the publication bias of each risk factor.

## 3. Results

### 3.1. Literature selection

**Figure 1.**
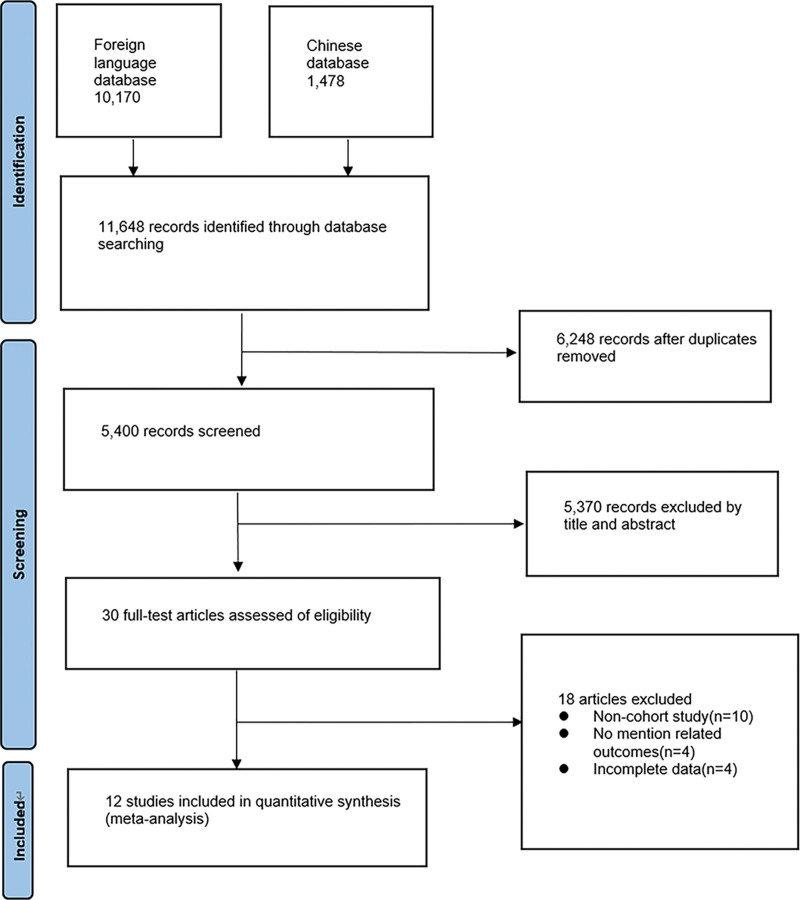
Study flow diagram.

A total of 11,648 related studies were obtained through systematic search, including 1478 in Chinese and 10,170 in English. Six thousand two hundred forty eight duplicate studies were removed and 5400 were retained. After reading the titles and abstracts, 5370 studies that did not meet the inclusion and exclusion criteria were excluded and 30 studies were retained. After further reading the whole study, 18 studies were removed, 10 articles were non-cohort studies, 4 articles did not mention relevant outcome indicators, and 4 articles had incomplete data. Eventually, 12 prospective cohort studies^[13–21]^ met the inclusion criteria. The flowchat is shown in Figure 1.

### 3.2. Study characteristics

The cohort studies included in the meta-analysis are shown in Table 1. The 12 prospective cohort studies included in this meta-analysis were published from 2005 to 2021, including 9 were published in international journals, and 3 were published in Chinese journals. Included studies were conducted in different countries, such as Spain, the United States, Argentina, India, etc. The sample size of this study ranged from 24 to 474, with a total of 1950 patients, of which there are patients in the ICU-AW group and patients in the non-ICU-AW group. The incidence of ICU-AW was 24% to 65% in 12 articles.

**Table 1:** Meta-analysis literature included in cohort study.

Study	Yr	Population	Country	Sample Size	ICU-AW incidence (%)	Risk factors	Diagnose	Age (mean ± SD, y)
Raurell et al	2021	ICU	Spain	474	58	1), 2), 31)	MRC-score	65 (54–74)
Nie et al	2019	ICU	China	142	48	1), 2), 3), 6), 8), 9), 11), 12)	MRC-score	58.6 ± 15.4
Zhanget al	2018	ICU	China	276	44	1), 2), 6), 8), 9), 10), 12)	MRC-score	59.41 ± 18.399
Donget et al	2017	ICU	China	256	44	1), 2), 6), 8), 9), 10), 12)	MRC-score	58.6 ± 17.9
Diaz et al	2017	ICU	Argentina	111	41	1), 2), 3), 5), 6), 10), 11), 13), 14), 32)	MRC-score	50.8 ± 17.15
Guptaet al	2016	ICU	India	100	37	3), 5), 6), 8), 9), 14), 29)	NCSs	63.5 ± 16.8
Anastasopoulos et al	2011	ICU	Greece	190	21	1), 2), 3), 4), 5), 6), 7), 13), 15), 16)	NCSs	65.5 ± 14.8
Sharshar et al	2010	ICU	France	86	45	2), 3), 17), 18), 19)	MRC-score	66 (51–78)
Weber-Carstens at al	2009	ICU	Germany	56	61	2), 20), 21), 22), 23), 24)	MRC-score	47.5 (32–60)
Nanas et al	2008	ICU	Greece	185	24	2), 3), 4), 5), 6), 7), 12), 13), 25), 26), 27), 28), 30)	MRC-score	55 ± 19
Khan et al	2006	ICU	America	48	65	1), 2), 3), 6)	NCSs	50.1 ± 16.3
Amaya-villar et al	2005	ICU	Spain	26	35	1), 3), 4), 5), 6), 8), 9), 10), 11), 14)	NCSs	63.4 ± 6.7

ICU = intensive care unit, ICU-AW = intensive care unit acquired weakness, MRC = Medical Research Council, NCSs = Nerve Conduction Studies. 1) Age; 2) Female; 3) Corticosteroids; 4) Aminoglucoside drugs; 5) Neuromuscular blockers; 6) APACHE II (Acute Physiology and Chronic Health Evaluation II) score; 7) SOFA (sepsis related organ failure assessment) score; 8) Mechanical ventilation days; 9) Length of ICU stay; 10) Infectious disease; 11) Sepsis; 12) Renal replacement therapy; 13) Hyperglycemia; 14) History of mechanical ventilation; 15) Septic shock; 16) Calcium ion concentration; 17) Sex hormones; 18) Insulin growth factor; 19) Thyroid stimulating hormone; 20) Norepinephrine; 21) SAPS (Simplified Acute Physiology Score) score; 22) SIRS (systemic inflammatory response syndrome); 23) Acute renal failure; 24) MODS (multiple organ dysfunction syndrome); 25) Vasoconstrictor drugs; 26) Gram-Negative bacteremia; 27) Pneumonia; 28) Parenteral nutrition; 29) Kidney replacement treatment days; 30) Incidence of hypoproteinemia; 31) Functional dependence before admission; 32) Delirium.

### 3.3. Assessment of studies quality

The quality assessment of these 12 studies is shown in Table 2. After NOS scale was used to evaluate the quality of the included studies, the score was 6 to 8, indicating that the included studies were of high quality.

**Table 2:** Results of literature quality evaluation.

The first author	Section	Comparability	Exposure	Total
Raurell	3	1	3	7
Nie	3	1	2	6
Zhang	3	1	3	7
Dong	3	1	2	6
Diaz	3	2	3	8
Gupta	3	1	2	6
Anastasopoulos	3	1	2	6
Sharshar	3	1	3	7
Weber-Carstens	3	1	2	6
Nanas	3	2	3	8
Khan	3	1	2	6
Amaya-villar	3	2	2	7

### 3.4. Risk factors for ICU-AW

**Figure 2.**
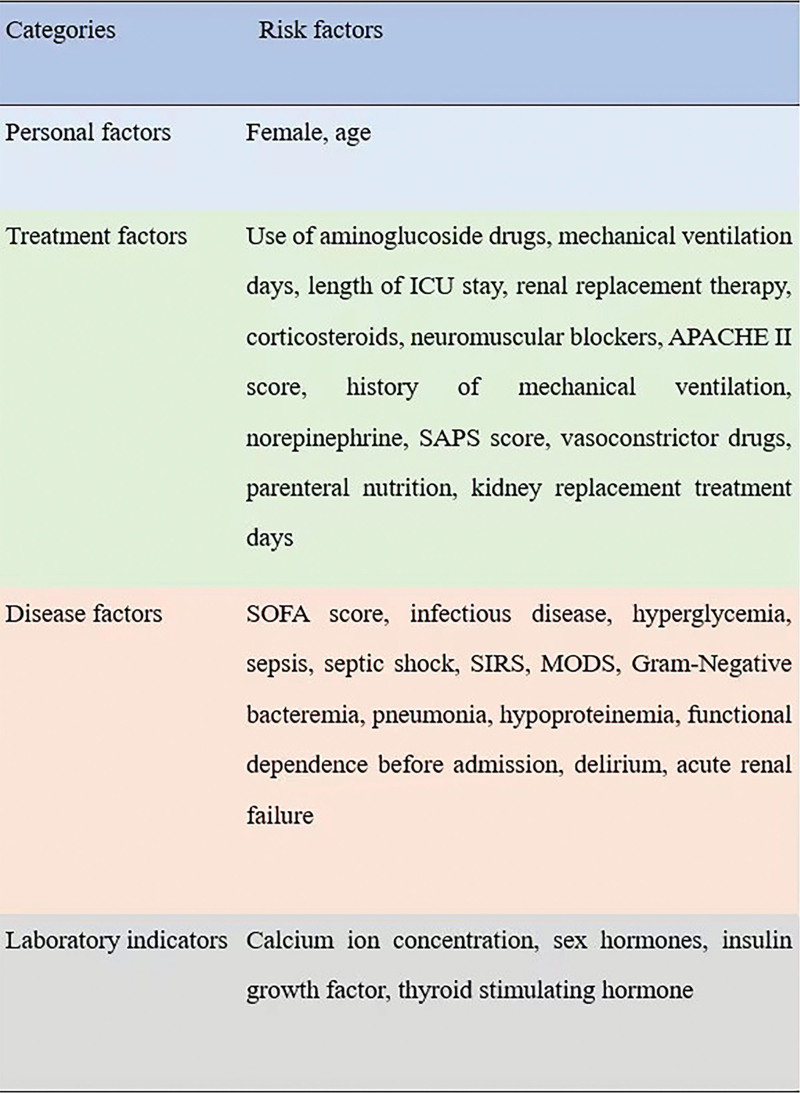
The related risk factors with ICU-AW. ICU-AW = intensive care unit acquired weakness.

The related risk factors are shown in Figure 2. Twelve articles reported a total of 32 risk factors, which could be divided into 4 categories: personal factors, therapeutic factors, disease factors, and laboratory indicators. Among them, statistically significant influencing factors were female, days of mechanical ventilation, age, length of ICU stay, infectious diseases, renal replacement therapy, aminoglycoside drug use, sepsis related organ failure assessment (SOFA) score.

#### 3.4.1. Female

**Figure 3.**
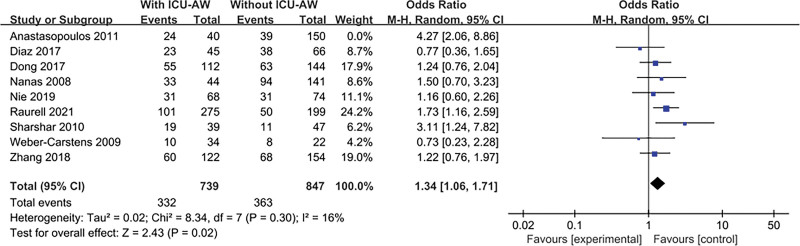
The meta-analysis results of female.

The result is shown in Figure 3. Ten literatures^[6,13,14,16,17,19,21–24]^ explored this content and tested for heterogeneity, *P* = .03, *I*^2^ = 53%, indicating heterogeneity. After sensitivity analysis, Anastasopoulos^[17]^ was the main source of heterogeneity. After excluding this study, there was a significant change in overall estimate, OR = 1.34, 95% CI:1.06 to 1.71; *P* = .02, There was no heterogeneity among studies, *I*^2^ = 16%, *P* = .30.

#### 3.4.2. Use of aminoglucosides

**Figure 4.**
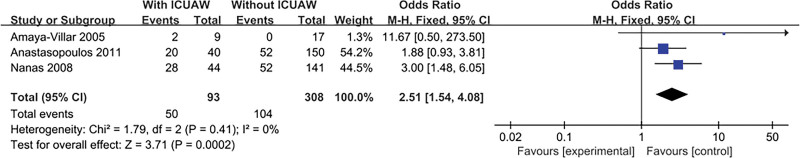
The meta-analysis results of using of aminoglucosides.

The result is shown in Figure 4. Three literatures^[17,20,21]^ discussed this content and tested for heterogeneity, *P* = .41, *I*^2^ = 0%, there was a good literature consistency, the fixed effects model was used, the combined effect was statistically significant, OR = 2.51, 95% CI: 1.54 to 4.08; *P* = .0002.

#### 3.4.3. Mechanical ventilation days

**Figure 5.**
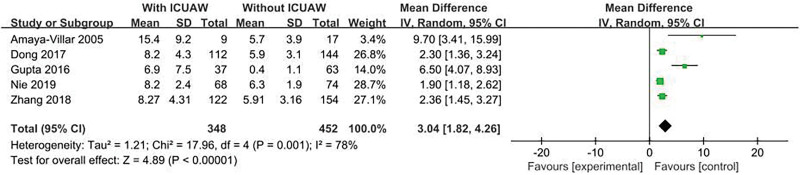
The meta-analysis results of mechanical ventilation days.

The result is shown in Figure 5. Five literatures^[15,20,22–24]^ explored this content and tested heterogeneity, *P* = .001, *I*^2^ = 78%, indicating heterogeneity. Therefore, sensitivity studies were conducted, excluding trials with relatively small sample size (n < 50), but there was no significant change in the overall estimate, OR = 2.73, 95% CI:1.65 to 3.80; *P* < .00001, however, significant heterogeneity was still observed, *I*^2^ = 76%, *P* = .005. Except for the experiment with the largest sample size, there was little change in overall estimates, OR = 3.62, 95% CI:1.79 to 5.44; *P* = .0001, significant heterogeneity still exists, *I*^2^ = 83%, *P* = .0004. The exclusion of either study did not change the combined estimate and heterogeneity (data not shown).

#### 3.4.4. Age

**Figure 6.**
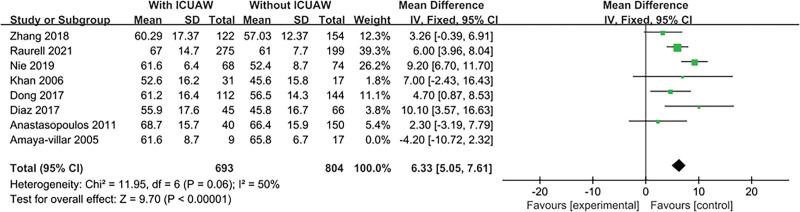
The meta-analysis results of age.

The result is shown in Figure 6. Eight literatures^[14,16,17,19,20,22–24]^ explored this content and tested for heterogeneity, *P* = .001, *I*^2^ = 78%, indicating heterogeneity. Therefore, we conducted a sensitivity study, excluding trials with relatively small sample sizes (n < 50), with a substantial change in overall estimates, OR = 6.33, 95% CI:5.05 to 7.61; *P* < .00001. There was no heterogeneity among studies, *I*^2^ = 50%, *P* = .06.

#### 3.4.5. Length of ICU stay

**Figure 7.**
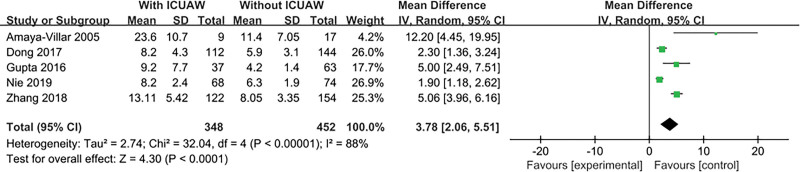
The meta-analysis results of length of ICU stay. ICU = intensive care unit.

The result is shown in Figure 7. Five literatures^[15,20,22–24]^ explored this content and tested for heterogeneity, *P* < .0001, *I*^2^ = 88%, there was heterogeneity in the literature. Therefore, we conducted a sensitivity study, excluding trials with relatively small sample sizes (n < 50), there was no substantial change in overall estimates, OR = 3.39, 95% CI:1.76 to 5.03; *P* < .0001. However, significant heterogeneity was still observed, *I*^2^ = 89%, *P* < .00001. Excluding the experiment with the largest sample size, there was still no substantial change in overall estimates, OR = 3.02, 95% CI:1.51 to 4.54; *P* < .0001, significant heterogeneity is still observed, *I*^2^ = 75%, *P* = .008. Excluding any one study will not change the combined estimates and heterogeneity (data not shown).

#### 3.4.6. Renal replacement therapy

**Figure 8.**
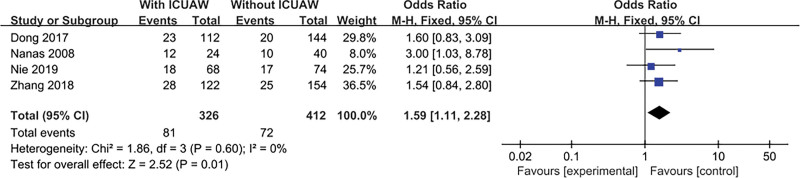
The meta-analysis results of renal replacement therapy.

The result is shown in Figure 8. Four literatures^[21–24]^ discussed this content and tested for heterogeneity, *P* = .60, *I*^2^ = 0%, the literature consistency was good, so we used the fixed effects model, and the combined effect was statistically significant, OR = 1.59, 95% CI: 1.11 to 2.28; *P* = .01.

#### 3.4.7. Infectious disease

**Figure 9.**
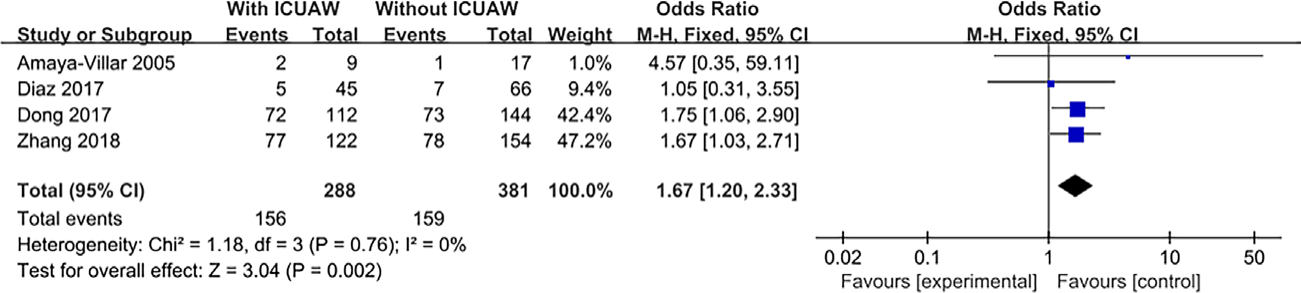
The meta-analysis results of infectious disease.

The result is shown in Figure 9. Four literatures^[14,20,22,24]^ discussed this content and tested for heterogeneity, *P* = .76, *I*^2^ = 0%, the literature consistency was good, the fixed effects model was used, and the combined effect was statistically significant, OR = 1.67, 95% CI: 1.20 to 2.33; *P* = .002.

#### 3.4.8. SOFA score

**Figure 10.**
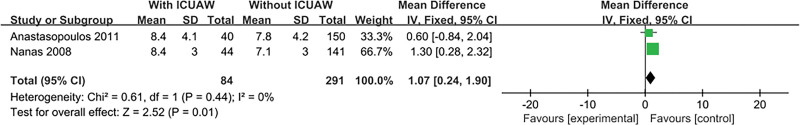
The meta-analysis results of SOFA score. SOFA = sepsis related organ failure assessment.

The result is shown in Figure 10. Two literatures^[17,21]^ discussed this content and tested for heterogeneity, *P* = .44, *I*^2^ = 0%, the literature consistency was good, the fixed effects model was used, and the combined effect was statistically significant, OR = 1.07, 95% CI: 0.24 to 1.90; *P* = .01.

#### 3.4.9. Use of corticosteroids

**Figure 11.**
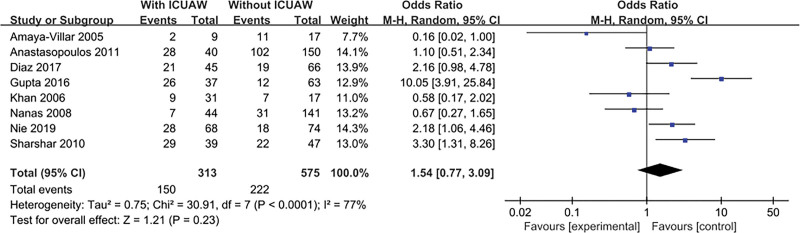
The meta-analysis results of using of Corticosteroids.

The result is shown in Figure 11. Eight literatures^[13–15,17,19–21,23]^ explored this content and tested for heterogeneity, *P* < .0001, *I*^2^ = 77%, there was a heterogeneity in the literature. Therefore, we conducted a sensitivity study, excluding trials with relatively small sample sizes (n < 50), there was no substantial change in overall estimates, OR = 1.86, 95% CI:0.96 to 3.62; *P* = .07, However, significant heterogeneity is still observed, *I*^2^ = 75%, *P* = .0005. Excluding the experiment with the largest sample size, there was no substantial change in overall estimates, OR = 1.60, 95% CI:0.71 to 3.59; *P* = .26, significant heterogeneity was still observed, *I*^2^ = 79%, *P* < .0001. Excluding any one study will not change the combined estimates and heterogeneity (data not shown).

#### 3.4.10. Use of neuromuscular blockers

**Figure 12.**
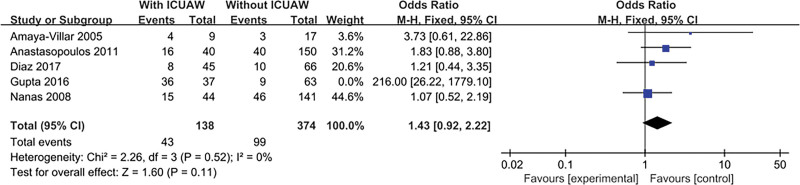
The meta-analysis results of using of neuromuscular blockers.

The result is shown in Figure 12. Five literatures^[14,15,17,20,21]^ discussed this content and tested for heterogeneity, *P* = .52, *I*^2^ = 0%, the literature consistency was good, the fixed effects model was used, and the combined effect was not statistically significant, OR = 1.43, 95% CI: 0.92 to 2.22; *P* = .11.·

#### 3.4.11. Sepsis

**Figure 13.**
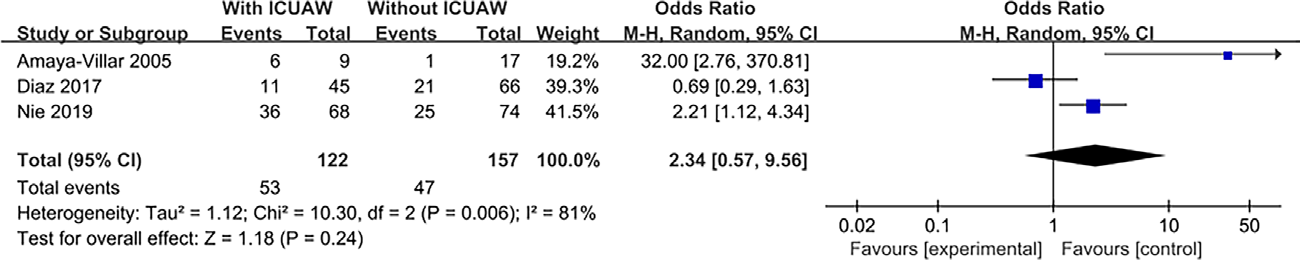
The meta-analysis results of sepsis.

The result is shown in Figure 13. Three literatures^[14,20,23]^ explored this content and tested for heterogeneity, *P* = .005, *I*^2^ = 81%, there was a heterogeneity in the literature. Therefore, we conducted a sensitivity study, excluding trials with relatively small sample sizes (n < 50), there was no substantial change in overall estimates, OR = 1.27, 95% CI:0.41 to 3.96; *P* = .67, However, significant heterogeneity is still observed, *I*^2^ = 77%, *P* = .04. Excluding the experiment with the largest sample size, there was no substantial change in overall estimates, OR = 3.95, 95% CI:0.09 to 172.68; *P* = .48, significant heterogeneity was still observed, *I*^2^ = 88%, *P* = .003. Excluding any one study will not change the combined estimates and heterogeneity (data not shown).

#### 3.4.12. Hyperglycemia

**Figure 14.**
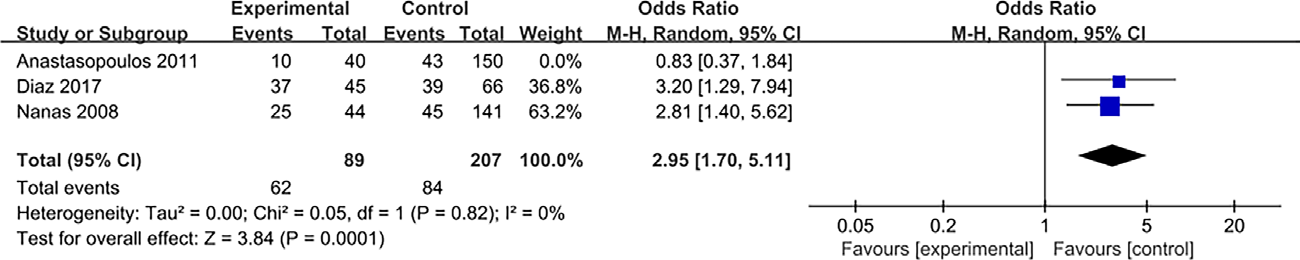
The meta-analysis results of hyperglycemia.

The result is shown in Figure 14. Three literatures^[14,17,21]^ explored this content and tested for heterogeneity, *P* = .04, *I*^2^ = 70%, there was a heterogeneity in the literature. Therefore, we conducted a sensitivity study, excluding trials with relatively small sample sizes (n < 50), there was no substantial change in overall estimates, OR = 1.55, 95% CI:0.47 to 5.12; *P* = .47, However, significant heterogeneity is still observed, *I*^2^ = 80%, *P* = .02. Excluding the experiment with the largest sample size, there was substantial change in overall estimates, OR = 2.95, 95% CI:1.70 to 5.11; *P* = .0001, There is no heterogeneity between studies, *I*^2^ = 0%, *P* = .82.

#### 3.4.13. Acute Physiology and Chronic Health Evaluation II (APACHE II) score

**Figure 15.**
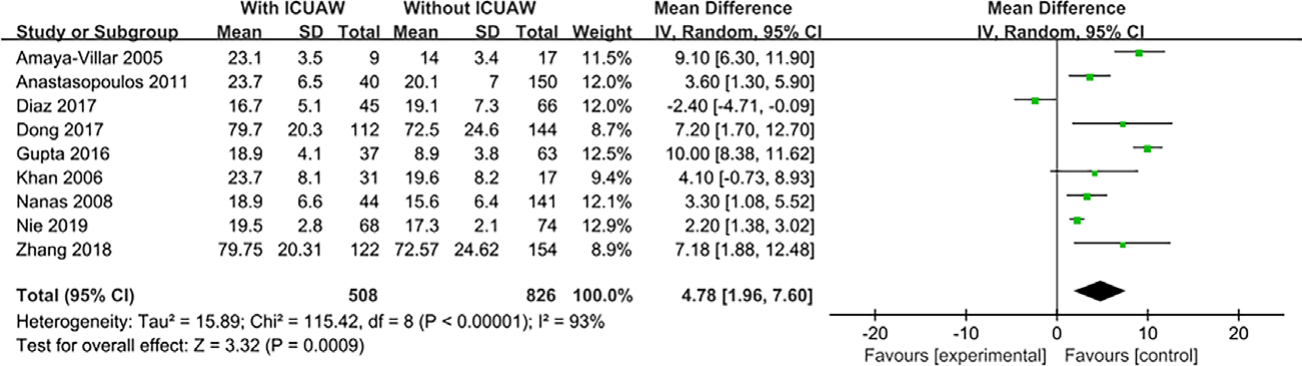
The meta-analysis results of APACHE II score. APACHE II = Acute Physiology and Chronic Health Evaluation II.

The result is shown in Figure 15. Nine literatures^[14,15,17,19–24]^ explored this content and tested for heterogeneity, *P* < .00001, *I*^2^ = 93%, The heterogeneity of the literature was obvious, so only describe. According to various literature studies and the occurrence of ICU-AW, APACHE II score is considered a risk factor that may cause ICU-AW.

## 4. Publication bias

In this study, the “female” index was included in the literature as a funnel plot, and the results showed that there was some asymmetry, possibly publication bias.

## 5. Discussion

Meta-analysis is the quantitative and scientific synthesis of research results. Since the term research synthesis and modern methods were first introduced in the 1970s, meta-analysis has a revolutionary impact in many areas of science, helping to establish evidence-based practice and resolve seemingly contradictory research findings.^[25]^ Acquired muscle weakness – a serious and costly medical complication – has become increasingly common in ICUS due to improvements in survival rates for critically ill patients over the past few decades.^[26]^ In recent years, ICU-AW has begun to attract people’s attention. But so far, the pathological mechanism of ICU-AW has not been fully understood, and there is still no particularly effective treatment. It has been suggested that ICU-AW is caused by a variety of factors work together. The relationship between some factors and the pathogenesis of ICU-AW and the pathogenesis are still unclear.^[8,9]^ The quality of the 12 articles included in this meta-analysis can be seen from the NOS scores. Most of the index data are were consistent during the analysis, laying a foundation for drawing reliable conclusions. We identified 32 risk factors for ICU-AW, few studies have shown the same risk factors. Thus, only 12 risk factors were used to calculate the size of the effect. To reach a consensus on the risk factors for ICU-AW, a large number of studies on individual risk factors are needed. In this study, the risk factors significantly associated with ICU-AW were female, days of mechanical ventilation, age, length of ICU stay, infectious diseases, renal replacement therapy, aminoglycoside use, SOFA score, and hyperglycemia. In conclusion, the risk factors identified in this study contribute to a better understanding of the pathogenesis and predictors of ICU-AW.

This systematic review and meta-analysis revealed that female is one of the important risk factors for ICU-AW and plays a vital role in the prediction of ICU-AW. Female patients are prone to ICU-AW. The ICU-AW risk prediction model constructed by Witteveen^[27]^ also included gender as a risk factor. A large number of studies have found that female is a risk factor for ICU-AW,^[28–30]^ but the mechanism of female susceptibility to ICU-AW remains unclear. Therefore, health care providers should pay more attention to female patients who are susceptible to ICU-AW.

This study found that age predisposed to ICU-AW. As we gradually enter an aging society, the average age of admission to the ICU is gradually increasing. The elderly (>60 years old) develop normal age-related muscle mass loss before admission to the ICU, this decrease in muscle mass is slow. However, in the severe cases, where systemic inflammatory responses, protein loss and anabolic stimulation are reduced, muscle loss of the elderly may be particularly rapid, which is the so-called muscle attenuation syndrome.^[17]^

This study found that mechanical ventilation was an independent risk factor for ICU-AW. It has been reported that normal people stay in bed strictly for 1 day, and their muscles decrease by 1%.^[31]^ The longer the mechanical ventilation days, the longer the patient needs to be immobilized. Studies have shown that when mechanical ventilation lasts for more than 5 days, the incidence of ICU-AW is 25% to 60%,^[32]^ further confirming that mechanical ventilation is an independent risk factor for ICU-AW. Aminoglycoside administration was also associated with ICU-AW.^[33–37]^ This finding is consistent with our research.

Due to the special nature of the ICU, patient has been in a state of immobility for a long time. Even healthy people, after fixed 4 hours every day, their muscles will decrease by 1% to 1.3% every day, and catabolism of patients with serious diseases may be aggravated, leading to the loss of muscle mass.^[29]^ Less than 10% of mechanically ventilated patients in the ICU reportedly do rehabilitation exercises in bed. Therefore, the longer the ICU stay in the hospital, the more likely ICU-AW will occur. In this study, length of ICU stay was an independent risk factor for ICU-AW. This is consistent with the findings of Hermans.^[5]^

An important finding of our study was the significant association between hyperglycemia during ICU stay and ICU-AW, which is consistent with the results of Hermans.^[5]^ Hyperglycemia can lead to decreased diaphragm function, and strict control of blood glucose can reduce the risk of critically ill polyneuropathy.^[17]^ This study suggests that the use of glucocorticoids and neuromuscular blockers are not independent risk factors for ICU-AW. The relationship between glucocorticoids and ICU-AW reported in a few prospective studies is not completely clear.^[30,38]^ Some studies have shown that glucocorticoid is a risk factor for ICU-AW and can lead to ICU-AW,^[39,40]^ while some studies have shown that low to moderate doses of glucocorticoids (For example, if patients with ARDS take methylprednisolone <1 mg/kg daily in the early stage and <2 mg/kg in the late stage) will not cause ICU-AW.^[5,41,42]^ The relationship between neuromuscular blockers and ICU-AW is not fully understood, and has varied from one study to another.^[5,43–46]^ Studies have shown that the occurrence of ICU-AW is related to the duration of neuromuscular blockers, and use of neuromuscular blockers for less than 48 hours does not increase the risk of ICU-AW.^[44,46,47]^ In a multicenter double-blind ACURASYS study conducted by Papazian et al, including 340 ARDS patients, the use of cis-atracurium for 48 hours did not increase the risk of ICU-AW.^[46]^ In addition, the occurrence of ICU-AW is related to the specific type, dose, and concomitant drugs (such as glucocorticoids) of neuromuscular blockers, which need to be further studied.^[38]^ Infection disease is also a risk factor for ICU-AW. Ischemia and inflammation may contribute to systemic infection in ICU patients. This weakness is associated with the release of inflammatory mediators. Inflammation can increase the permeability of capillaries, and its toxicity is more likely to release neuromuscular complications.

The relevant studies we reviewed in this study were found to have publication bias. This may be because we only selected studies published in academic journals, and studies with negligible results may not have been published. Second, the publication bias found in this study may be due to the limited overlap of risk factors between studies. Therefore, further research is needed to accumulate evidence on each risk factor.

## 6. Limitations of this study

This study has a little limitations. First of all, we only reviewed prospective cohort studies, so other types of related studies may be excluded. The studies included in this meta-analysis were all from published literature. Gray literature is not included, which may have potential publication bias. Secondly, different types of studies have differences in sample size and case selection, which may lead to heterogeneity among studies and have a certain impact on research results. Finally, due to the limited number of studies, it is impossible to calculate the impact of all risk factors. Whereas, this article makes an important contribution to understanding the current situation by integrating research on ICU-AW risk factors and identifying areas that need further research. It is recommended that more high-quality, multi-center, and large-sample original studies for verification in the future, and conduct a more comprehensive and scientific evaluation of the influencing factors of ICU-AW, so as to provide early warning for the clinial practice.

## 7. Conclusion

Through this systematic review and meta-analysis, the risk factors for ICU-AW were identified as follows: female, days of mechanical ventilation days, age, length of ICU stay, infectious diseases, renal replacement therapy, aminoglycoside drug use, SOFA score, and hyperglycemia. However, the evidence for the predictive value of glucocorticoids, neuromuscular blockers, and sepsis is insufficient and needs to be validated by more high-quality studies in the future. Based on the existing evidence, it is suggested that clinical medical staff should pay attention to the impact of ICU-AW on patients, improve the awareness of early warning, actively screen and identify high-risk groups, and classify risks. The occurrence and development of ICU-AW is a dynamic process, medical staff should take comprehensive intervention measures as soon as possible to effectively prevent ICU-AW.

## Acknowledgements

We would like to thank the support of the medical staff in the intensive care units of the Second Affiliated Hospital of Harbin Medical University.

## Author contributions

**Conceptualization:** Zi Yang, Faying Wang, Zeyu Peng.

**Data curation:** Zi Yang.

**Formal analysis:** Zi Yang.

**Funding acquisition:** Yuying Fan.

**Investigation:** Zi Yang.

**Methodology:** Zi Yang.

**Resources:** Zeyu Peng.

**Software:** Xiaohui Wang.

**Supervision:** Xiaohui Wang.

**Validation:** Xiaohui Wang.

**Visualization:** Xiaohui Wang.

**Writing – original draft:** Faying Wang.

**Writing – review & editing:** Faying Wang, Zeyu Peng.
